# Calcium Oxalate Crystallization: Influence of pH, Energy Input, and Supersaturation Ratio on the Synthesis of Artificial Kidney Stones

**DOI:** 10.1021/acsomega.1c03938

**Published:** 2021-10-01

**Authors:** Helen Werner, Shalmali Bapat, Michael Schobesberger, Doris Segets, Sebastian P. Schwaminger

**Affiliations:** † Bioseparation Engineering Group, Department of Mechanical Engineering, Technical University of Munich, 85748 Garching, Germany; ‡ Process Technology for Electrochemical Functional Materials, Institute for Combustion and Gas Dynamics—Reactive Fluids (IVG-RF), University of Duisburg-Essen (UDE), 47057 Duisburg, Germany; § Center for Nanointegration Duisburg-Essen (CENIDE), 47057 Duisburg, Germany; ∥ Department of Chemical Engineering, Massachusetts Institute of Technology, Cambridge, Massachusetts 02139, United States

## Abstract

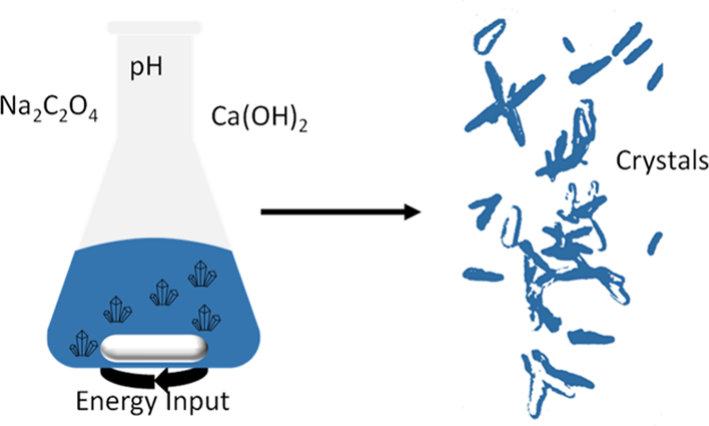

The removal of kidney stones can lead to small residual fragments remaining in the human body. Residual stone fragments can act as seeds for kidney stone crystallization and may necessitate another intervention. Therefore, it is important to create a consistent model with a particle size comparable to the range of kidney stone fragments. Thus, the size-determining parameters such as supersaturation ratio, energy input, and pH value are examined. The batch crystallizations were performed with supersaturation ratios between 5.07 and 6.12. The compositions of the dried samples were analyzed with Raman spectroscopy, infrared spectroscopy, and X-ray diffraction (XRD). The samples were identified as calcium oxalate monohydrate with spectroscopic analysis, while calcium oxalate dihydrate being the most prominent crystalline species at all supersaturation ratios for the investigated conditions. The aggregate size, obtained with analytical centrifugation, varied between 2.9 and 4.3 μm, while the crystallite domain size, obtained from XRD, varied from 40 to 61 nm. Our results indicate that particle sizes increase with increasing supersaturation, energy input, and pH. All syntheses yield a high particle heterogeneity and represent an ideal basis for reference materials of small kidney stone fragments. These results will help better understand and control the crystallization of calcium oxalate and the aggregation of such pseudopolymorphs.

## 1 Introduction

Calcium oxalate is a mineral of the oxalate group^1^ and occurs naturally in humans, in plants, and in various industrial processes.^2−6^ In the pulp and paper industry and in the production process of sugar, it is important to suppress the precipitation of calcium oxalate.^4,7^

Calcium oxalate can also be precipitated in humans and cause the formation of kidney stones (urolithiasis) in the urogenital tract. Due to social and climate-related changes, the number of kidney stone diseases is constantly increasing.^8^ With regard to Germany, this results in four million people suffering from urolithiasis at the beginning of the 21st century.^9^ The composition of kidney stones is variable and depends on the living conditions of the patient; however, most kidney stones consist of calcium oxalate.^10^ The latter are formed by the supersaturation of urine with calcium and oxalate ions, whereby the so-called principle of heterogeneous nucleation is present.^9,11^ The urine is saturated with ions in the metastable range, and the crystallization is triggered by promoters such as crystal nuclei or cell detritus.^9^ Particle formation can be divided into two overarching processes: nucleation/growth and subsequent aggregation.^12−14^ During nucleation, nuclei are formed, growing into larger aggregates through collisions. Nucleation can be triggered by de novo synthesized fragments (primary nucleation) or fragments present in the solution (secondary nucleation).^14^

Currently, several therapy forms are applied using a high-power laser system to destroy kidney stones, enabling them to pass the urogenital tract. The laser system is often combined with a small basket to grip bigger fragments which is known as retrograde intrarenal surgery.^16^ A novel therapy approach employs a frequent irradiation of holmium or thulium laser to dust or pop-dust the whole stone.^17,18^ The different therapy approaches bring various risks: among others, the residue of clinically insignificant residual fragments (CIRFs) with a size of <1 mm.^15,16,18^ The importance of CIRFs is controversial, and new studies suggest that they may be a catalyst for new stones, leading to additional treatment.^16,19,20^ Thus, it is important for the further development of the existing therapies and the risks of CIRFs to create a realistic and consistent model.^21^

Recent studies of calcium oxalate crystallization in a batch system are investigating, inter alia, the amount of water of crystallization, depending on the supersaturation ratio.^22,23^ It could be deduced that calcium oxalate monohydrate (COM) or whewellite was the predominant crystal system.^24^ Furthermore, different promoters,^25^ inhibitors,^26,27^ or processes were investigated in the production of calcium oxalate.^28,29^

Our aim is to control the crystallization of calcium oxalate and to obtain heterogeneous fragments which can be used as reference materials for kidney stone calculi. The focus of this study is on the properties and how the fragments behave in external fields such as centrifugation fields. In the following, the crystallization of calcium oxalate is performed in a batch process, and afterward, the dried sample is analyzed in the powder form. The influence of the supersaturation ratio, the stirrer speed, and the pH value on the composition of the sample, as well as the particle size distribution, is examined to control the particle size of calcium oxalate crystals. The identity of the sample is determined by Fourier transform infrared (FT-IR) spectroscopy, Raman spectroscopy, and powder X-ray diffraction (XRD). The hydrodynamic diameter of the aggregates is determined by analytical centrifugation (AC).

## 2 Results and Discussion

### 2.1 Influence of the Supersaturation Ratio

Powder samples were analyzed with Raman spectroscopy, attenuated total reflectance (ATR) FT-IR spectroscopy, and optical microscopy. The microscopy images (Figures S1–S3) confirm the samples’ tetragonal crystalline structures.

**Figure 1:**
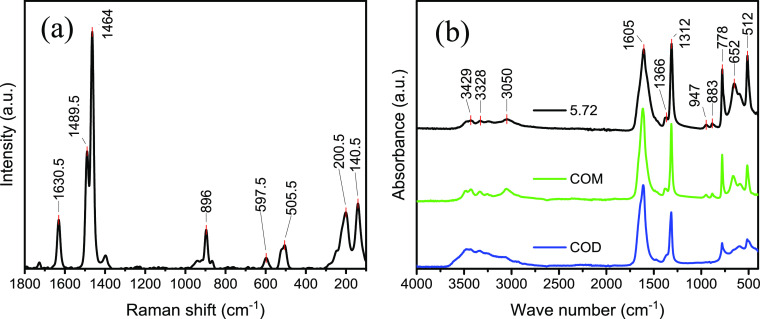
(a) Raman spectrum of the powder form of the sample crystallized at a supersaturation ratio of 5.72, measured with a 488 nm laser and a power of 4 mW. (b) FT-IR spectrum of the powder form of the sample crystallized at a supersaturation ratio of 5.72, measured in a range between 400 and 4000 cm^–1^. The ATR–IR spectra of COD and COM from the RUFF database have been used as a reference.

Figure 1a shows the characteristic peaks for calcium oxalate at 1630, 1488, 1464, 896, and 504 cm^–1^ for the sample crystallized at a supersaturation ratio of 5.80 (results for different supersaturation ratios are summarized in Figures S4 and S5).^36,37^ The asymmetric carboxylate stretch vibrations correspond to the band at 1630 cm^–1^, while the symmetric carboxylate stretch vibrations correspond to the bands at 1490 and 1464 cm^–1^.^38^ The peak at 896 cm^–1^ is assigned to the C–C vibration, and the peak at 504 cm^–1^ corresponds to the O–C–O in-plane bending.^36,38,39^ While all spectra indicate the presence of COM being the most prominent species for all obtained Raman spectra, the presence of other hydrates such as calcium oxalate dihydrate (COD) seems likely. Especially, the ratio of the vibrations at 1490 and 1464 cm^–1^ is affected by the different supersaturation concentrations. There is a tendency of decreasing ratios of the peaks 1490 to 1464 cm^–1^ which can be assigned to changes in either hydration or shape differences for these crystals.

Figure 1b illustrates the FT-IR spectrum of the sample crystallized at a supersaturation ratio of 5.72. The spectrum shows characteristic peaks for calcium oxalate at 3429, 3328, 3049, 1605, 1365, 1312, 947, 883, 777, 652, and 512 cm^–1^ (results for different supersaturation ratios are summarized in Figures S6 and S7).^38,40^ The peaks between 3400 and 3000 cm^–1^ can be assigned to the stretching vibrations of the hydroxyl groups and show the typical behavior of COM precipitates.^38,39^ The symmetric and asymmetric vibrations of the carbonyl group can be observed at 1312, 1365, and 1605 cm^–1^.^38^

The IR spectrum shows additional peaks at a wavenumber of 947 cm^–1^ due to the C–O stretching vibrations.^39^ The peak at 777 cm^–1^ is caused by C–C stretching vibrations, and the peak at 652 cm^–1^ is caused by O–H out-of-plane bending vibration.^39^ The O–C–O plane bending vibration corresponds to the bands at 512 cm^–1^. The shifts in the peaks compared to the values of calcium oxalate in the FT-IR spectra arise from physisorbed water.^24,40,41^

Here, significant amounts of physisorbed water and water of crystallization can be observed in the IR spectrum. COM consists of two oxalate units, one planar unit and the other one coiled with a smaller C–C distance. In contrast, the structure of COD shows two equivalent planar units with a smaller distance between the C–C bond.^36^ The spectra indicate the presence of COD and COM with COM being the most prominent species in all spectra (Figures S6 and S7).^38,42^

XRD was further used to determine the accurate lattice parameters and thus the amount of water in the crystal structure. In addition, the crystallite domain size was calculated with the Scherrer equation (eq 3). Rietveld refinement yields a tetragonal structure which corresponds to COD;^43^ however, COM and its triclinic structure can be observed as well (eq 2).^38^

Moreover, amounts of calcium oxalate trihydrate (COT) or caoxite can be observed in the diffractograms as well.^44^ However, in all diffractograms, COD is by far the most prevalent species which can be observed in the diffractograms.

The calculated lattice parameters are *a* = 6.82, *b* = 7.98, and *c* = 12.92 Å. The observed diffraction data indicate the presence of crystalline phases in all dried samples (Figures S8 and S9).^43^

Supersaturation ratios are related to the solubility product of COD, shown in Table 1. Additionally, the crystallite domain size, which is calculated with the Scherrer equation (eq 3), is presented in Table 1. In the first step of the crystallization process, crystallites are formed. Their sizes depend on the supersaturation ratio and vary between 34 and 37 nm. The varying diameters, which are based on the Scherrer analysis of the 111 reflex, can be affected by different crystal shapes of COD, which affect the XRD pattern.^40,42^ However, as here only four supersaturation ratios were studied, it is a challenge to unambiguously verify an increasing crystallite domain size or changing particle shape with increasing supersaturation ratio, as reported in the literature.^22,23,45^

**Table 1:** Crystallite Domain Sizes Derived from XRD Data (Figure S7) Depending on the Supersaturation

CaCl_2_ [mmol L^–1^]	Na_2_C_2_O_4_ [mmol L^–1^]	ionic strength I [mmol L^–1^]	activity coefficient *f*_±_	supersaturation ratio COD [—]	supersaturation ratio COM [—]	crystallite domain size [nm]
2	1.2	9.6	0.69	5.07	6.45	34
5	1.2	18.6	0.61	5.72	7.10	35
7.5	1.2	26.1	0.56	5.96	7.34	37
10	1.2	33.6	0.53	6.12	7.50	36

**Figure 2:**
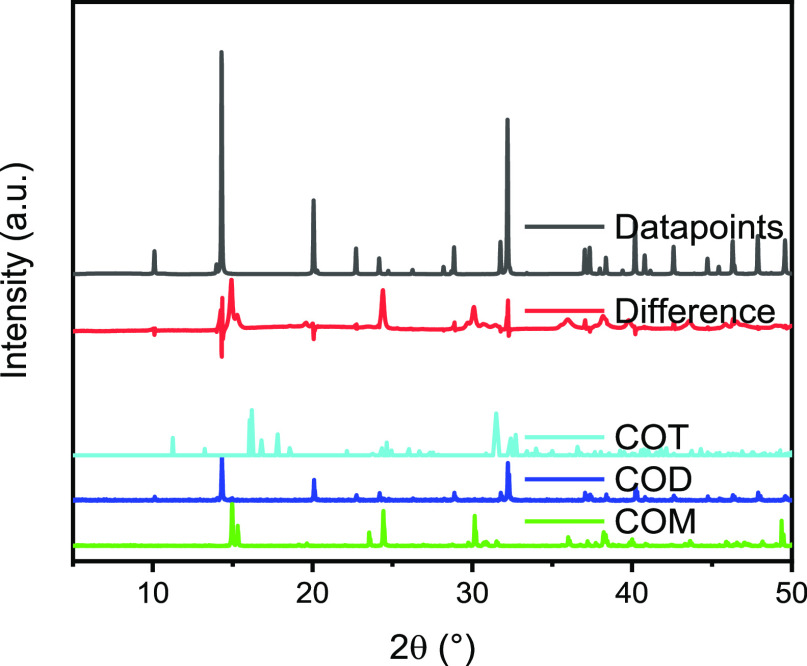
Diffractogram and Rietveld refinement of the diffractogram of the powder sample crystallized at a supersaturation ratio of 5.72 irradiated with Cu Kα_1_. Data points correspond to a refinement of COD, and the difference corresponds to COM. The references of COM (whewellite), COD (weddellite), and COT (caoxite) from the RUFF database are shown.

Spectroscopic analysis shows no influence of the supersaturation ratio between 5.07 and 6.12 on the formation of mono-, di-, or trihydrates since the spectra do not diverge significantly (Figures S4–S7). In IR and Raman spectra, the OH stretch vibrations between 3450 and 3050 cm^–1^ correspond to a mixture of hydroxide vibrations, which mainly contain COM and COD.^38,39,42^ In the IR spectra, the ratio of the prominent band at 1605 cm^–1^ corresponding to C=O stretch vibrations maintains a stable ratio to the band at 1312 cm^–1^, corresponding to a C–C vibration (Figures S6 and S7).^38^ Hence, COD is formed as a main crystalline component at all supersaturations investigated. As a result of the latter and due to the applied temperature, the formation of COD is preferred.^43,46,47^ The content of COM is less than 10% and represents the main component, which is not refined as COD (Figure 2).^48^ The dihydrate structure is preferred for higher ionic concentrations and supersaturations.^22,23,49^ In addition, the solution is filtered after an incubation time of 20 min. For the complete transformation of COD to its monohydrate structure, an incubation time of 24 h is needed.^48,50^ However, the drying process can affect the hydration state of the pseudopolymorphs.^38^ Furthermore, the monohydrate is the most stable form which might be precipitated around the COD crystallites, which explains their presence and dominance in the IR and Raman spectra.^22,38,51^ Additionally, the transformation of trihydrate to dihydrate, caoxite, which has been observed in the XRD as well, during the incubation time is possible.^49^

**Figure 3:**
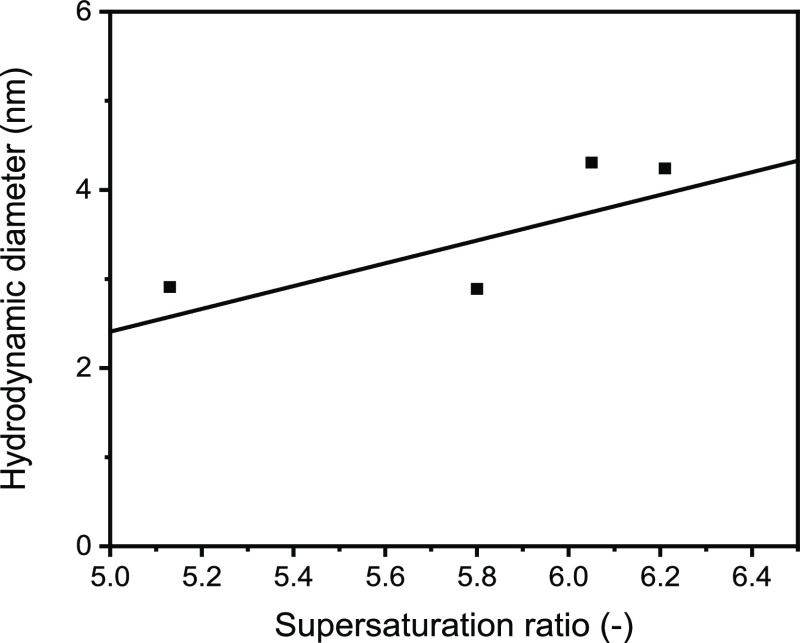
Hydrodynamic diameter measured by AC as a function of the supersaturation ratio.

The experiments focusing on the influence of the supersaturation ratio were performed at a pH of 5 as the risk of crystallization of calcium oxalate in urine is the highest at a pH between 4.5 and 5.5.^52^ The determination of the hydrodynamic particle diameters was performed based on AC data and Stokes’ law.^53^Figure 3 shows the correlation between the hydrodynamic diameter and the supersaturation ratio, determined in a centrifugation field. In brief, hydrodynamic diameters between 2.9 and 4.3 μm were obtained (Figure S10). The significantly larger hydrodynamic diameters compared to the crystallite domain sizes can be explained by aggregation.^25,54^ The large standard deviation of up to ±3 μm can be explained by the heterogeneity of the particle sizes, which exhibit a broad particle size distribution.

Another challenge to characterize the particles is their asymmetry, which can be observed in the microscopy pictures and is well illustrated in the cumulative size distributions of length and width (Figures S12 and S13). The length of oxalate particles varies from 5 to 100 μm, while the width is in a narrow range between 1 and 10 μm.

**Figure 4:**
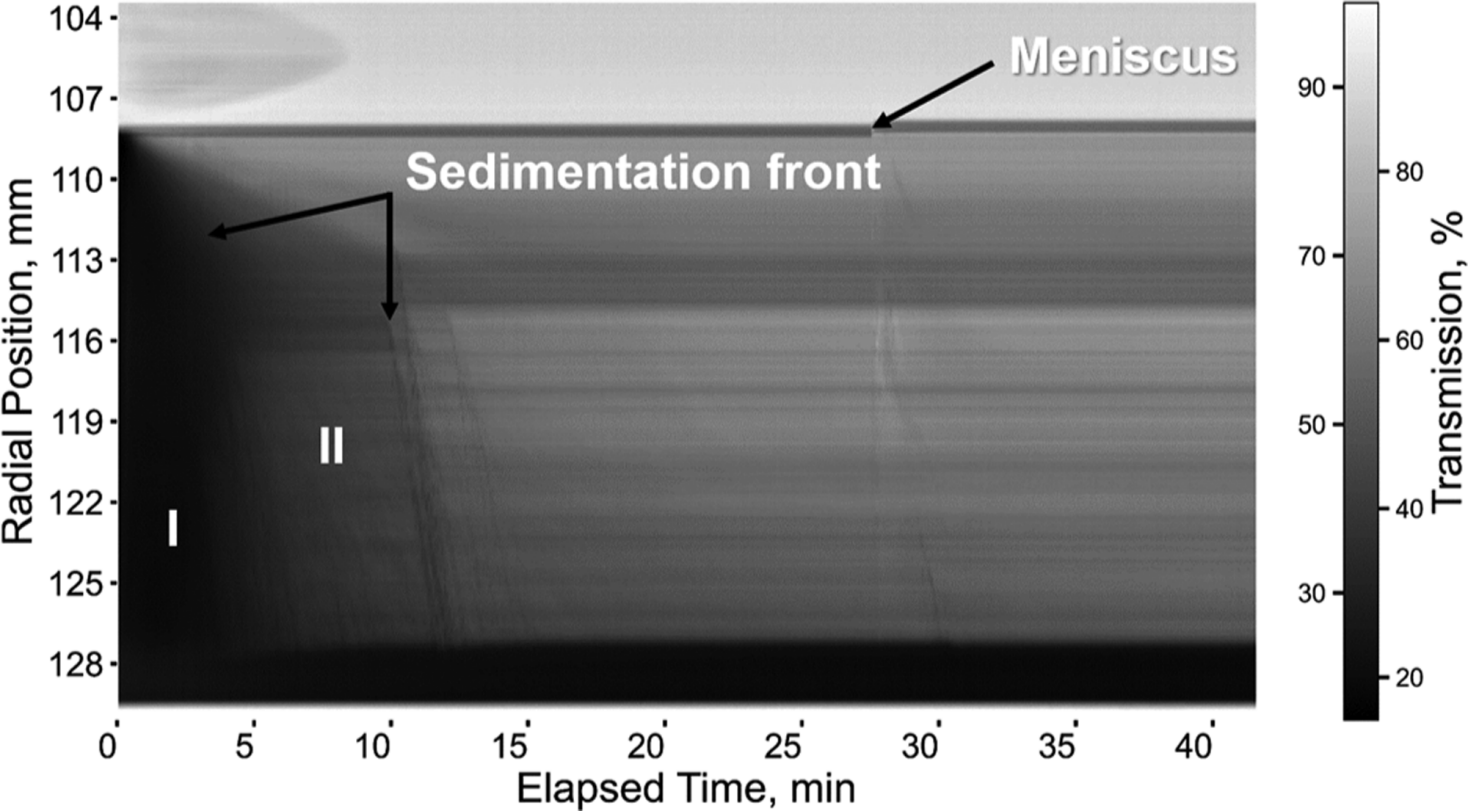
Exemplary transmittogram of the sample crystallized at a supersaturation ratio of 5.96. A sucrose concentration of 25% was used as the continuous phase for the experiment to slow down sedimentation. The meniscus and sedimentation fronts are marked by black arrows. I and II indicate sedimentation fronts of larger and smaller aggregates, respectively.

The broad distribution in the sample is further confirmed by the transmittogram, as depicted by Figure 4. The *x*-axis represents the time elapse from the start of centrifugation. The *y*-axis represents the sample filling height spanning from a radial distance of ∼103 mm to a radial distance of ∼129 mm from the rotor center. For orientation, the meniscus is clearly seen around 108 mm. The heterogeneity due to broad size distributions is manifested by sedimentation fronts annotated in Figure 4. The first sedimentation front indicates the existence of larger fragments which settle in about 6 min, while the second sedimentation front confirms the existence of comparatively smaller aggregates which settle in ∼12 min. Some fragments remain dispersed in the continuous phase, as shown by the darker shade of gray between 12 and 32 min. The figure provides a granular look at the time duration where only the first ∼40 min of the experiment is shown. The complete settling takes place after ∼40 min, where the liquid becomes clearer, as indicated by the lighter shade of gray (see Figure S14). The transmittograms visualize the inhomogeneities of the synthesized oxalate fragments very well (Figures 4, S14, and S15). The same trend of optical densities over time and thus similar sedimentation fronts can be observed for sample 2 (supersaturation 5.72) and sample 3 (supersaturation 6.05) (Figures 3 and S15). Also, samples 1 and 4 with the supersaturations 5.07 and 6.12 show similar transmittograms with less pronounced sedimentation fronts occurring at earlier elapsed times (Figures S14 and S15). Interestingly, the sedimentation time is neither in agreement with the crystallite domain sizes obtained from XRD (Table 1) nor with the analysis of the median size obtained from AC (Figure 3). However, the sedimentation times in the transmittograms show a similar trend to the size analysis of microscopy images, underlining the complexity in the size characterization of the generated particles. An important conclusion here is that—similar to the assessment of colloidal surface properties^100−56^—for the full picture, a variety of characteristic sizes should be analyzed. Samples 2 and 3 demonstrate the smallest median size (5 and 9 μm) and width (2 and 2 μm) and the narrowest average equivalent diameters, respectively (Figures S12 and S13). Samples 1 and 4 show different transmittograms, with a significantly smaller range of dark shades in the beginning. This is in line with the microscopy images that also demonstrated larger median particle sizes (18 and 20 μm) and widths (4 and 4 μm) (Figures S12 and S13).

As a summary, AC analysis between supersaturation ratios of 5.07 and 6.12 shows an exponential relationship for oxalate fragments’ size dependence on supersaturation. This can be explained as follows: since the nucleation rate and thus the formation of nuclei are exponentially dependent on the supersaturation, the ratio can be used to influence the density of the nuclei and the rate of addition of the building blocks. As the supersaturation ratio increases, more nuclei are formed, which grow into larger aggregates. In addition, the supersaturation ratio acts as a concentration gradient and generates a transport of the nuclei through the suspension.^14^

The width of the particle size distribution and the standard deviation of the hydrodynamic diameter with increasing supersaturation ratio is shown in Figure S10. All standard deviations are very large. This means that the synthesized particles exhibit a high polydispersity, which is in good agreement with the transmittograms corresponding to these particles and the microscopy images (Figures S12–S15). Thus, the supersaturation influences the aggregation process. In comparison, other crystallization systems do not show any influence of supersaturation on the primary nucleation, e.g. by different number of nuclei and/or changing surface properties as will be discussed later.^57^ The recommended supersaturation ratio results in smaller particle sizes. This enables the production of particles with a more uniform size that better represents the size range of CIRFs.

As mentioned above, this supersaturation ratio produced with a calcium chloride concentration of 5 mmol L^–1^ and a sodium oxalate concentration of 1.2 mmol L^–1^ was used for further investigations on the influence of the energy input and pH value. The ζ potential is a measure for colloidal stability and surface charge of oxalate particles. The latter determines the repulsive forces between the particles and therefore the aggregation process. Furthermore, ζ potential indicates the surface charge and surface composition, which determine how particles grow and how adhesives and other particulates can be attached to particles. Hence, the ζ potential is important to make estimations on the electrochemical double layer forming around the particles and the resulting effects on adsorption of metal ions to the oxalate particles.^58^ The ζ potential is strongly dependent on the surface structure and composition and on the ionic strength of the solution.^59^ In human kidneys, the ionic strength is usually isotonic and the pH is slightly acidic.^60^ The following Table 2 shows the measured ζ potential and the standard error of the mean depending on the supersaturation. All particles show a negative ζ potential between −15 and −23 mV at pH 7, which is in good agreement with COM particles in a similar size range.^59^ This means that the particles should not tend to aggregate fast but are also not completely colloidally stable. The ζ potential is closest to zero for the smallest particles (supersaturation ratio 5.72).

**Table 2:** ζ Potential Depending on Supersaturation and Standard Deviation (SD)

supersaturation ratio [—]	ζ potential [mV]	SD [mV]
5.06	–22.7	±3.1
5.72	–15.3	±2.1
5.96	–23.0	±4.9
6.12	–19.5	±5.2

The results show for all supersaturation ratios negative ζ potentials with a mean value of −20.1 mV. The deviations between the measured values are the result of different crystal shapes of COD due to the varying supersaturation ratio.^61^ Some crystal shapes consist of a higher density of positively charged calcium ions, resulting in a less negative ζ potential.^40^ Since the particles are very heterogeneous, the ζ potentials cannot be used as an indicator for the aggregation of oxalate particles.

### 2.2 Influence of the Energy Input and pH Value

The process of crystallization is not only limited by the nucleation rate and the number of nuclei but also by their transport.^22^ In an unstirred suspension, the primary particles diffuse in the direction of the concentration gradient and accumulate to form larger aggregates. The concentration gradient is generated by the supersaturation. Increased stirring reduces diffusion limitation and decreases the agglomeration efficiency but increases the breakage of aggregates.^54^ Furthermore, the increased energy input and thus a higher Reynolds number accelerate the transport of the primary particles.^14^ Regarding crystal–crystal collisions, the surface charge is another important parameter which determines whether particles are colloidally dispersed and stable or aggregate. The surface charge and the resulting repulsive forces due to electrostatic interactions are directly influenced by the pH value.^14^ Therefore, in the following experiments, the influence of both parameters on the particle diameter are considered. Additionally, the crystallite domain size is calculated as described above. The energy input is defined by the dimensionless Reynolds number and Newton number, which makes it possible to be compared independently of the experimental setup.

Table 3 shows the crystallite domain size based on the XRD (Figure S5) depending on the pH value and the energy input. The crystallite domain size ranges between 18 and 43 nm, and the crystal phase mainly consists of COD.^38^ It can be assumed that by changing the two parameters, the crystal structure and hence the crystallite domain size are affected.^40^ A larger average crystallite domain size can be achieved with a higher pH. However, the energy input does not have a major influence on this parameter.

**Table 3:** Crystallite Domain Sizes Derived from XRD Data Depending on the Energy Input

#	pH	Reynolds number	Newton number	crystallite domain size [nm]
1	5	732	2.00	18
2	5	1147	1.95	25
3	5	1615	1.85	33
4	9	732	2.00	36
5	9	1147	1.95	30
6	9	1615	1.85	43

**Figure 5:**
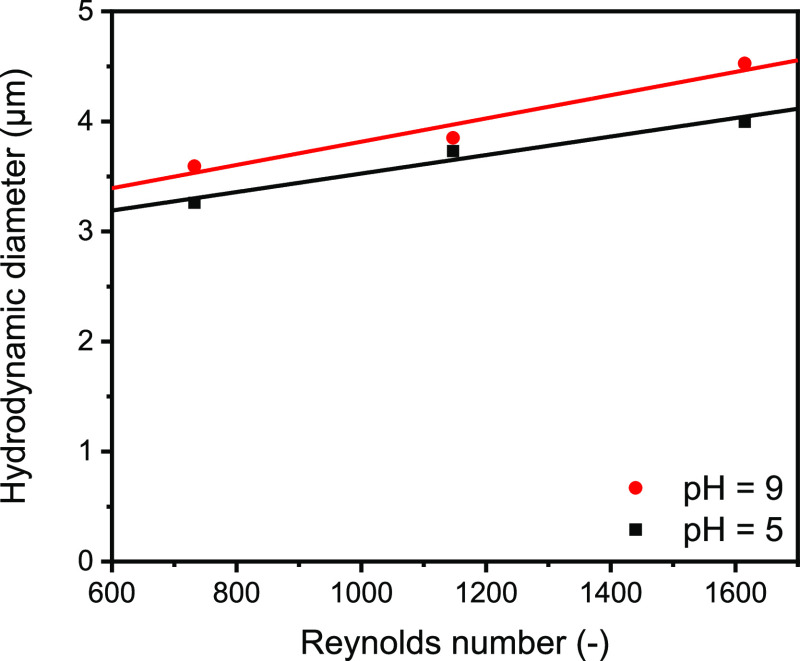
Hydrodynamic diameter as a function of the Reynolds number in a range between *Re* = 732.4 and *Re* = 1614.

**Figure 6:**
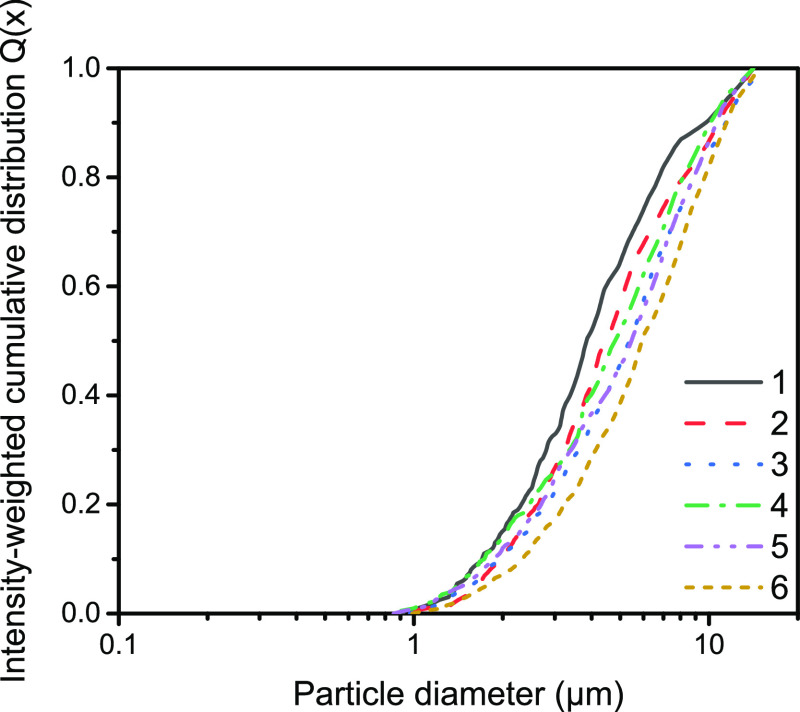
Intensity-weighted cumulative size distribution *Q*(*x*) with varying energy input and pH. Numbers 1–6 correspond to the sample code used in Table 3.

In Figure 5, the particle diameter at pH values of 5 and 9 is presented as a function of the Reynolds number. The particle diameter increases with increasing Reynolds number from 3.0 to 4 μm at pH 5 and from 3.3 to 4.5 μm at pH 9. As described in the Experimental Section,^13,35^ the Newton number was determined with a stirrer-dependent performance characteristic and amounts 2, 1.95, and 1.85 in a transitional flow regime for Reynolds numbers of 732, 1147, and 1615, respectively. The heterogeneity of the fragments was further verified optically from transmittograms derived from AC analysis corresponding to these experiments (Figures 6 and S11). The microscopy images indicate a different order of particle sizes since samples 1 and 4, with the lowest Reynolds numbers, show the largest particle sizes (25 and 19 μm) and widths (5 and 4 μm) (Figures S12 and S13). The AC analysis verifies the microscopy images since the settling here is faster than for other samples (Figure S11). However, for all samples, the hydrodynamic diameter which is derived from sedimentation analysis is in the same range (Figures S11 and S13).

As shown in Figure 5, there is a linear relationship between the Reynolds number and the average value of the colloid diameter at pH 5 and 9. In line with the findings on the effect of the supersaturation ratio, this is explained as follows: with an increasing energy input, the collision of nuclei is increasing and therefore the diameter increases too. At both pH values, the diffusion limitation is the predominant limiting parameter for the growth of aggregates at a supersaturation ratio of 5.72.^14,25^

Finally, the intensity-weighted cumulative distributions of different energy inputs at pH 5 and 9 during the synthesis of oxalate fragments are shown in Figure 6. These distributions agree very well with the analysis of the microscopy images (Figure S13) and support the assumption that with increasing energy input and pH, the particle size distribution shifts to larger distributions, too.^54^ This effect can be ascribed by an increasing aggregation rate due to more collisions at higher stirring rates.^54^ The discrepancy between the aggregation size at pH 5 and pH 9 can be explained with the faster nucleation rate at lower pH. A higher number of nuclei leads to smaller aggregates during the aggregation process.^52,54^ During the crystallization process, calcium oxalate precipitates at the beginning and remains on the bottom of the reaction vessel at low speeds for the whole reaction time.^62^ For these crystals, the growth rate is slowed down and they do not move freely in the suspension to form aggregates. As a result, the system is still not in equilibrium. This assumption is supported by the identification of COD as main species in all samples (Figures S4–S9). The latter is formed in case the system is kept unbalanced, for example, by insufficient mixing and discrepancies in local concentrations.^43^ Furthermore, a higher stirring rate and better mixing conditions lead to higher shares of COD.^22,23^ Hence, the stirring rate and pH influence the aggregation to a greater extent than the supersaturation during the crystallization process.

For the studies of the energy input, a similar behavior can be observed for all particles synthesized. All particles clearly indicate COM as the most prominent species in IR and Raman spectra, while COD is the most prominent phase in the diffractograms (Figures S5, S7, and S9).^38,39,44^ In the diffractograms, only COD and COM can be observed, while no indication of COT is visible. The phase purity of weddellite is higher than 80% for all samples. Higher stirring rates led to higher phase purity of COD in the diffractograms, which is in good agreement with other studies on the influence of the stirring rate on the polymorphic behavior of calcium oxalate hydrates.^22^ We did not observe a significant dependence of the spectra on the stirring rate of the experiments. Here, the influence of the supersaturation was more prominent than the stirring rate.

All particles synthesized can be used as a model for CIRFs since they represent very small calcium oxalate aggregates which can also be generated during the dusting procedure in kidney stone treatment.^16,63,64^

## 3 Conclusions

The crystallization of calcium oxalate was investigated with the aim of creating model particles that allow the examination of CIRFs. We wanted to create standard CIRFs to ensure that their composition is independent of the patient’s medical history and that the procedure can be performed with standard laboratory equipment. Crystallization was carried out at different supersaturation ratios followed by the analysis of the crystal structure and water content of crystallization. ATR–FT-IR and Raman spectroscopy analysis indicate that calcium oxalate was present at all supersaturation ratios. The analysis of the lattice parameters obtained from XRD shows that COD is the most prominent crystalline species which is present at all supersaturation ratios. However, COM is more prominent in all spectroscopy studies, which indicates the presence of COM precipitates alongside COD crystallites. The crystallite domain size of the synthesized particles ranged from 25 to 61 nm. The variation of the supersaturation ratio between 5.07 and 6.12 showed no change in the composition of the generated samples, and therefore, COD and COM were present at the measured supersaturation ratios in this range.

Also, the aggregation and therefore the ζ potential of the synthesized COD/COM particles were in the focus of our study. To investigate the aggregation, AC was conducted. The ζ potentials of all synthesized fragments are in the range of −15 to −25 mV. Analysis of transmittograms yields very fast settling aggregates in the centrifugal fields. This behavior can be observed with AC particle size distributions and with transmittograms and is supported by the analysis of microscopy images. The supersaturation ratio, pH value, and energy input determine the colloid diameter of oxalate fragments. Especially, the pH value determines the average particle diameter. At a pH value of 9, the largest colloids are formed; however, at both pH values investigated, the particle size can be influenced through the stirring speed. The risk of developing calcium oxalate stones in a kidney is the highest at a pH value of 5. Thus, we considered the particles synthesized at pH 5 to create a realistic model for the synthesis of calcium oxalate crystals as a reference material for kidney stone fragments.^52^ Hence, we were able to demonstrate a potentially scalable synthesis of calcium oxalate crystals with heterogeneous particle size distributions and pseudopolymorphic behavior which will serve as a reference material for kidney stone fragments in the future.

## 4 Experimental Section

### 4.1 Materials

Sodium oxalate solution was produced with sodium hydroxide (Carl Roth GmbH + Co. KG) and oxalic acid (Merck KGaA). Calcium chloride solution was obtained from AppliChem GmbH. Deionized water was used for all solutions. The cellulose filter membranes were obtained from Th. Geyer GmbH & Co. KG with a pore size of 0.2 μm.

### 4.2 Methods

For the supersaturation experiments, the sodium oxalate concentration was adjusted to 1.2 mmol L^–1^; for all experiments, the calcium chloride concentration was varied between 2 and 10 mmol L^–1^. Calcium oxalate was prepared by rapidly mixing 250 mL of sodium oxalate and 250 mL of calcium chloride solution. The initial pH value of both solutions was adjusted to 5 with hydrochloric acid. The solutions were poured into a stirring tank reactor and homogeneously stirred with a magnetic stirring bar. The stirring speed was adjusted to 80 rpm. Crystallization was conducted at room temperature for 20 min. The pH value was measured at the beginning and at the end of the crystallization. The resulting suspension was filtered through a cellulose filter membrane with a pore size of 0.2 μm by means of vacuum, and the filter paper was dried for 1 h in a drying cabinet at 80 °C.

In experiments to investigate the influence of the energy input and the pH value, the sodium oxalate concentration was adjusted to 1.2 mmol L^–1^ and a calcium chloride concentration of 5 mmol L^–1^ was used.

Crystallization was initiated in a stirring tank reactor with a total volume of 500 mL, into which 250 mL of sodium oxalate solution was provided and 250 mL of calcium chloride solution was added. The solution was mixed with varying stirring speeds at transitional stirring behavior (50, 80, and 110 rpm). The experiments were conducted at room temperature for 20 min and at pH values of 5 and 9, which were adjusted with hydrochloric acid and sodium hydroxide, respectively. Again, the suspension was separated following the crystallization using a cellulose filter membrane with a pore size of 0.2 μm, and the filter paper was dried for 1 h at 80 °C in a drying cabinet.

The composition of the crystallization product was determined with ATR–FT-IR spectroscopy, Raman spectroscopy, and XRD. The sample was analyzed in the powder form. The ATR–FT-IR spectra were accumulated 24 times with an Alpha 2 (Bruker Optics GmbH) in the wavenumber range between 400 and 4000 cm^–1^ and a spectral resolution of 4 cm^–1^. Raman spectroscopy was performed with a SENTERRA (Bruker Optics GmbH) using a 488 nm laser at a power of 4 mW and a spectral resolution of 15 cm^–1^. Both FTIR spectra and Raman spectra have been baseline-corrected with a concave rubberband correction. The powder XRD measurements were performed with Mo Kα_1_ radiation with a wavelength of 0.709 Å, and the overnight measurement was performed with Cu Kα_1_ with a wavelength of 1.541 Å (STOE Stadi P). The 2θ range was 2–60° with a step width of 0.015° for the Mo Kα_1_ X-ray source and 5–80° for the Cu Kα_1_ X-ray source with a step width of 0.015°. Microscopic images are made with an AXIO Observer 7 from Zeiss with differential interference contrast and an Axiocam 506 mono. The particle size distribution was analyzed using an AC device LUMiSizer 611 (LUM GmbH, Berlin, Germany). All measurements were carried out at a wavelength of 865 nm. Measurements were conducted at room temperature in a water solution and at 10 °C with a sucrose solution (25%). The higher viscosity of the latter was chosen to better visualize the sedimentation process, and the lower temperature was chosen to reduce Brownian motion. For the ζ potential characterization, a solid concentration of 1 g L^–1^ was used, and the measurement was performed with a Delsa Nano C (Beckman Coulter Inc., USA). The pH of the sample was measured afterward with a pH meter from SI-Analytics.

The supersaturation ratio *S* is introduced as a parameter for the crystallization of calcium oxalate. For this purpose, the supersaturation ratio *S* was calculated from the natural logarithm of the product of activities divided by the solubility product of COD or weddellite *K*_sp_ (see eq 1). The concentration in the solution was calculated from the product of the activities of the calcium and the oxalate ions. The solubility product of COD is 6.76 × 10^–9^ mol^2^ L^–2^, and the solubility product of COM is 1.7 × 10^–9^ mol^2^ L^–2^.^30^ The activities were determined by the product of concentrations and activity coefficients *f*_±_ according to Davies with the charge number *z* and the ionic strength *I* (see eq 2).^31^ The activity coefficients are listed in Table 1.
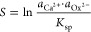
1
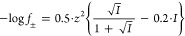
2

The spectral alignment of FT-IR and Raman spectra was performed with the software OriginPro (OriginLab). The analysis of the diffractograms and the Rietveld refinement was conducted with the software Match!. The corresponding calculation of the crystallite domain size D was performed according to the Scherrer equation with the form factor *K* = 0.89, the wavelength λ, half the maximum intensity HB, and the diffraction angle θ^32^
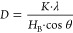
3

The particle size distributions were calculated using the LUMiSizer software SepView 6.4 in accordance with ISO 13318-2.^33^ In brief, the raw data for the transmission profiles were accessed from the software. Scientific libraries in Python language were used to construct transmittograms.^34^ A transmittogram is an image developed by plotting a heatmap from transmission data captured by AC. The *x*- and *y*-axes of the plot represent the elapsed centrifugation time and the radial position of the sample, which spans between ∼103 and ∼130 mm. Gray values between 0 and 255 are assigned to the instantaneous transmission represented by a colorbar. Thus, the color gradient indicates changes in the transmission or the concentration of the solute. The darkest color implies the lowest transmission values corresponding to the highest concentration, which is the case at the beginning of the experiment. As the experiment progresses, due to the separation process in the centrifugal field, the transmission increases, and thus, the brightest color represents the highest transmission, signifying the lowest concentration. A detailed description for the construction of transmittograms has been reported elsewhere.^34^

The energy input was characterized by the dimensionless Reynolds number *Re* with the stirrer diameter *d*, the stirrer speed *N*_*i*_, the fluid density ρ, and the fluid viscosity μ.^13^
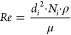
4

The Newton number *Ne* was determined with a stirrer-dependent performance characteristic.^35^

## Supporting Information Available

Microscopy images of oxalate crystals; Raman spectra of oxalate crystals; ATR IR spectra of oxalate crystals; X-ray diffractograms of oxalate crystals; hydrodynamic diameters of synthesized particles; particle sizes obtained from microscopy analysis; and transmittograms of oxalate particles (PDF)
