# Supplementary material for: Calcium Oxalate Crystallization: Influence of pH, Energy Input, and Supersaturation Ratio on the Synthesis of Artificial Kidney Stones
Source: ACS Omega. 2021 Oct 1;6(40):26566–74. doi: 10.1021/acsomega.1c03938 (PMC8515601; doi:10.1021/acsomega.1c03938)
Supplement: Supplementary file 1 — ao1c03938_si_001.pdf [file ao1c03938_si_001.pdf]

Supporting Information

Calcium Oxalate Crystallization: Influence of pH, Energy Input and Supersaturation Ratio on the Synthesis of Artificial Kidney Stones

Helen Werner,[†] *Shalmali Bapat,*[‡] *Michael Schobesberger,*[†] *Doris Segets,*[‡,§] *Sebastian P. Schwaminger,**[†,⊥]

[†]Bioseparation Engineering Group, Department of Mechanical Engineering, Technical University of Munich, 85748 Garching, Germany

[‡]Process Technology for Electrochemical Functional Materials, Institute for Combustion and Gas Dynamics – Reactive Fluids (IVG-RF), University of Duisburg-Essen (UDE), Duisburg, Germany

[§]Center for Nanointegration Duisburg-Essen (CENIDE), Duisburg, Germany

[⊥]Department of Chemical Engineering, Massachusetts Institute of Technology, Cambridge, 02139, MA, United States

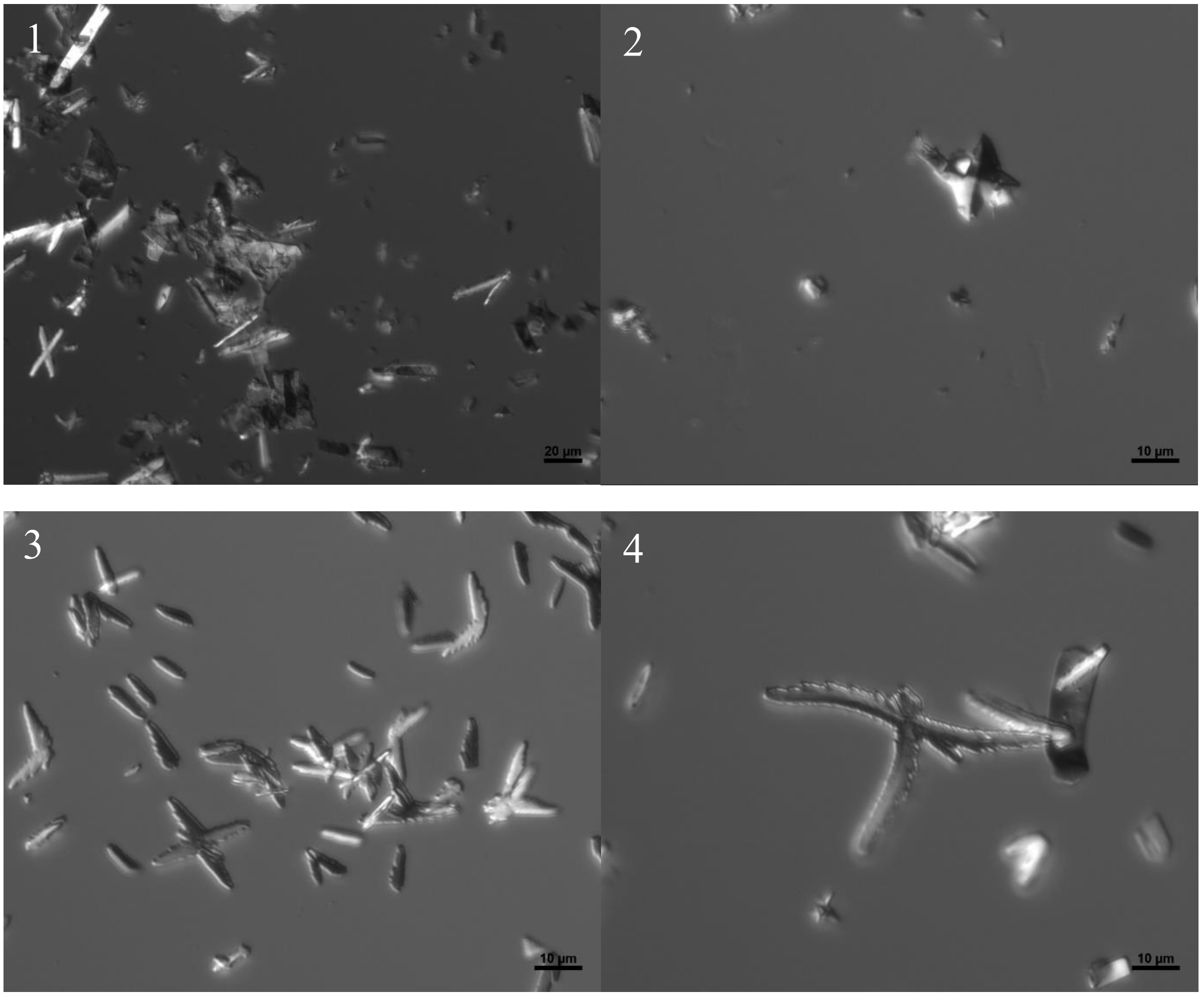

Figure S1. Differential interference contrast microscopy images. Crystallized samples at different supersaturation ratios (5.07 – 6.12) are shown. They are numbered from 1-4 in the same order as Table 1 and 2.

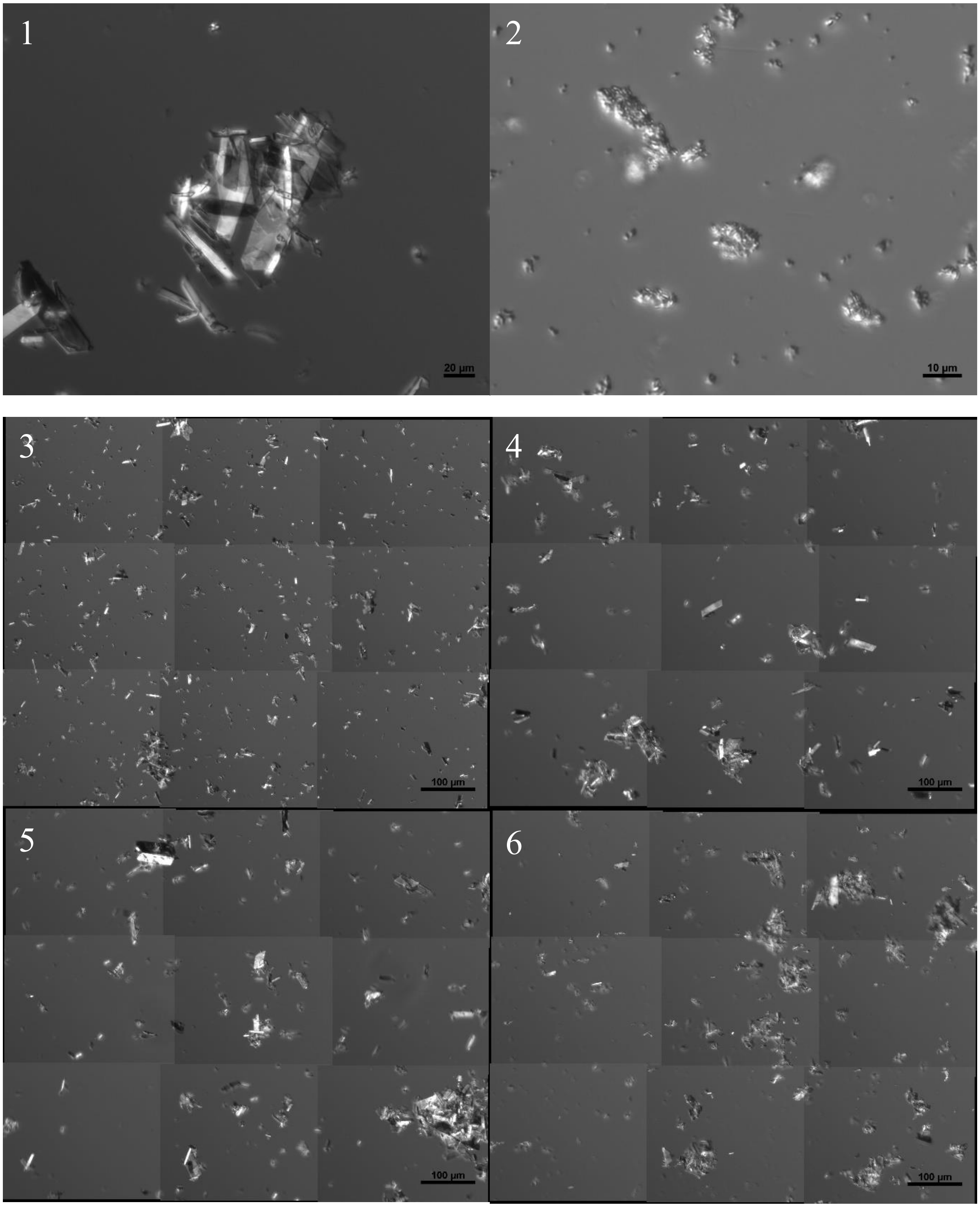

Figure S2. Light microscopy images. Crystallized samples at different Reynolds numbers and pH are shown. Samples are numbered according to Table 3 (pH 5, 732; pH 5, 1147; pH5, 1615; pH 9, 732; pH 9, 1147; pH9, 1615).

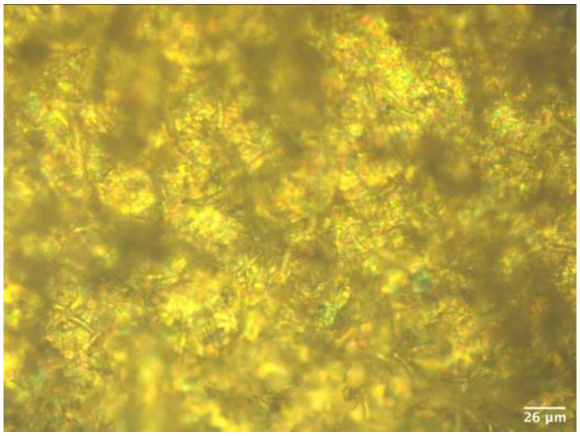

Figure S3. Image taken with a microscope of the dried samples crystallized with a supersaturation ratio 5.72.

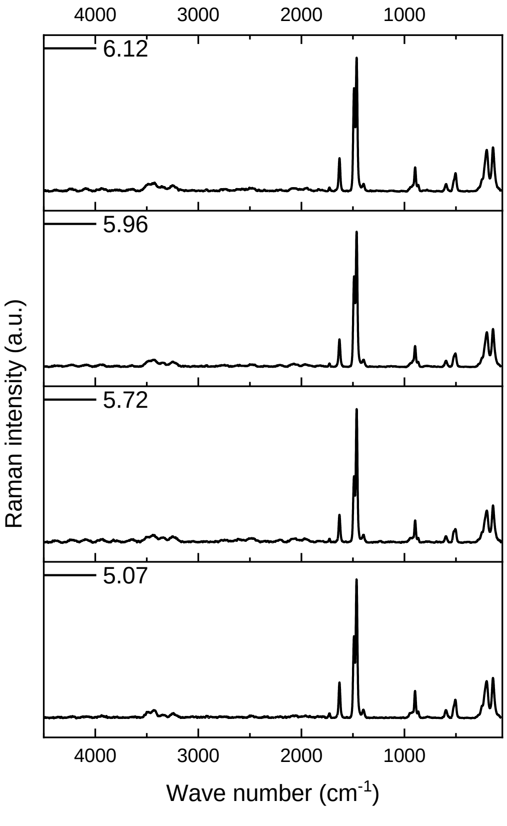

Figure S4. Raman spectra of samples crystallized at different supersaturation ratios (5.13 – 6.21), measured with a 488 nm laser and a power of 4 mW.

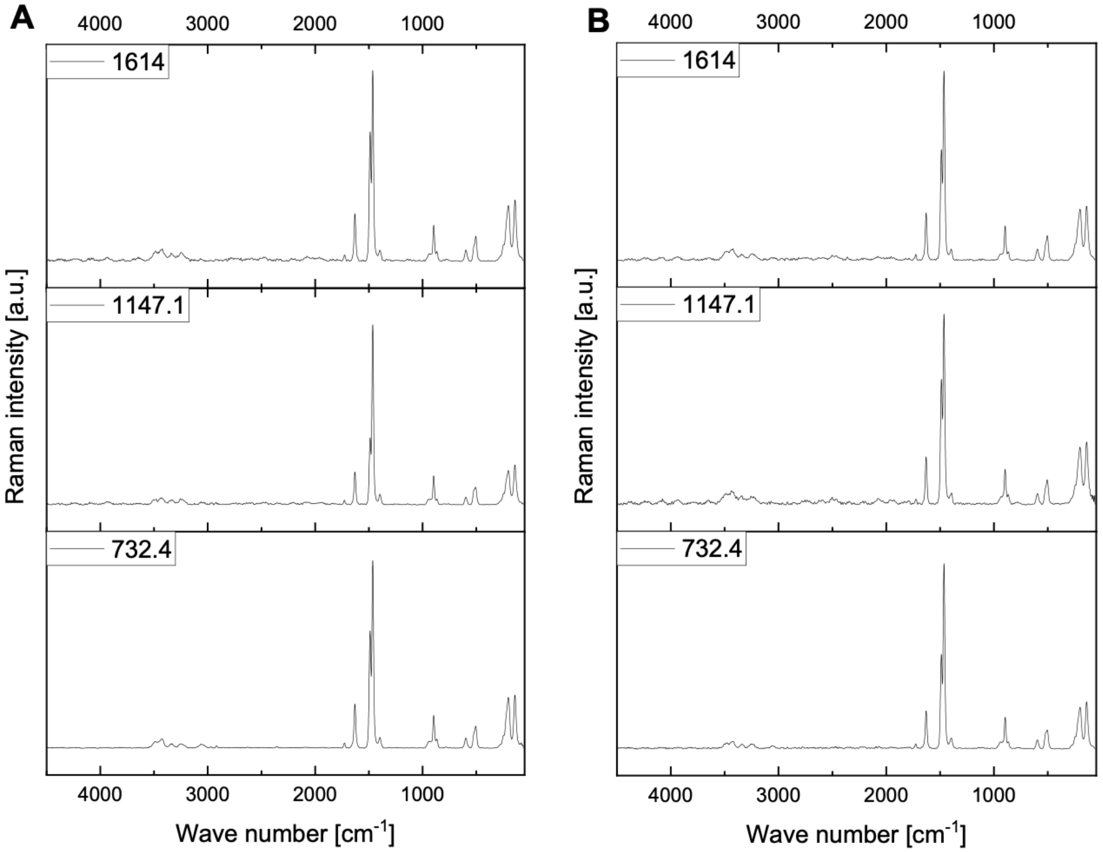

Figure S5. Raman spectra of the samples crystallized at a supersaturation ratio 5.72, at different Reynolds numbers (732, 1147, 1614), measured with a 488 nm laser and a power of 4 mW; A: at pH 5; B: at pH 9.

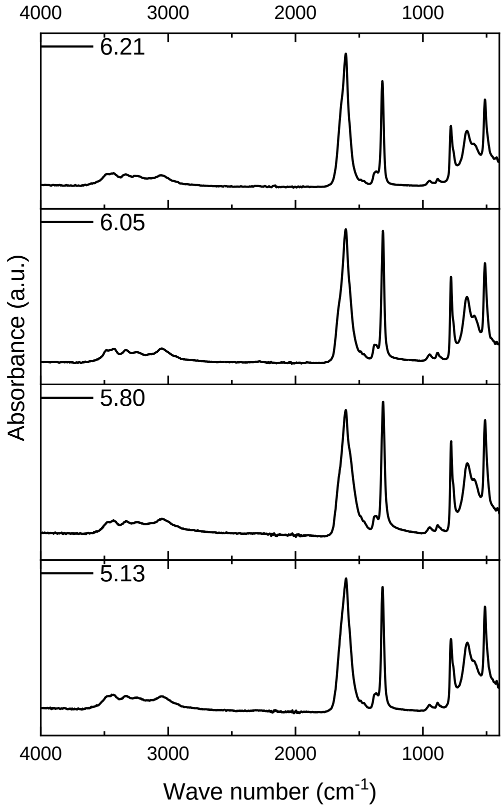

Figure S6. ATR FT-IR spectra of the samples crystallized at different supersaturation ratios (5.07 – 6.12); measured in a range between 400 – 4000 cm^{-1}.

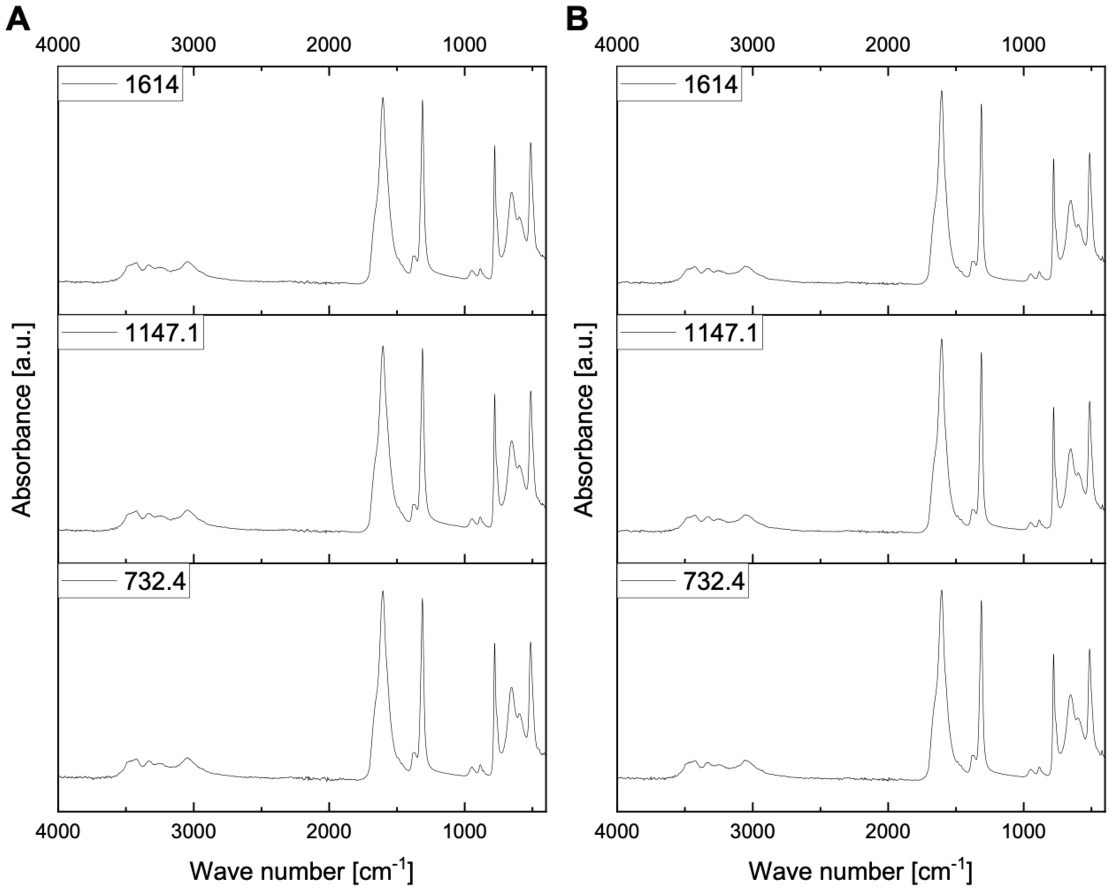

Figure S7. ATR FT-IR spectra of the samples crystallized at a supersaturation ratio of 5.72, at different Reynolds numbers (732, 1147, 1614), measured in a range between 400 – 4000 cm^{-1}; A: at pH 5; B: at pH 9.

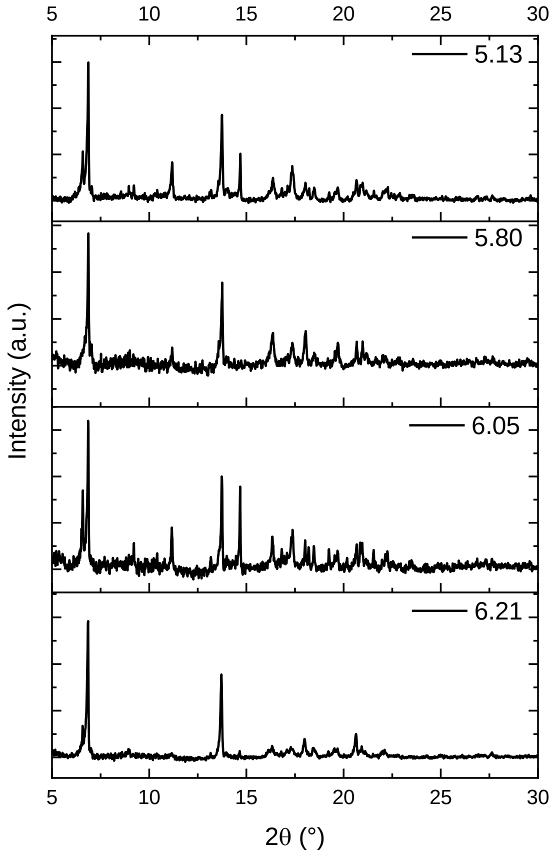

Figure S8. XRD patterns of the crystallized samples at different supersaturation ratios (5.07 – 6.12).

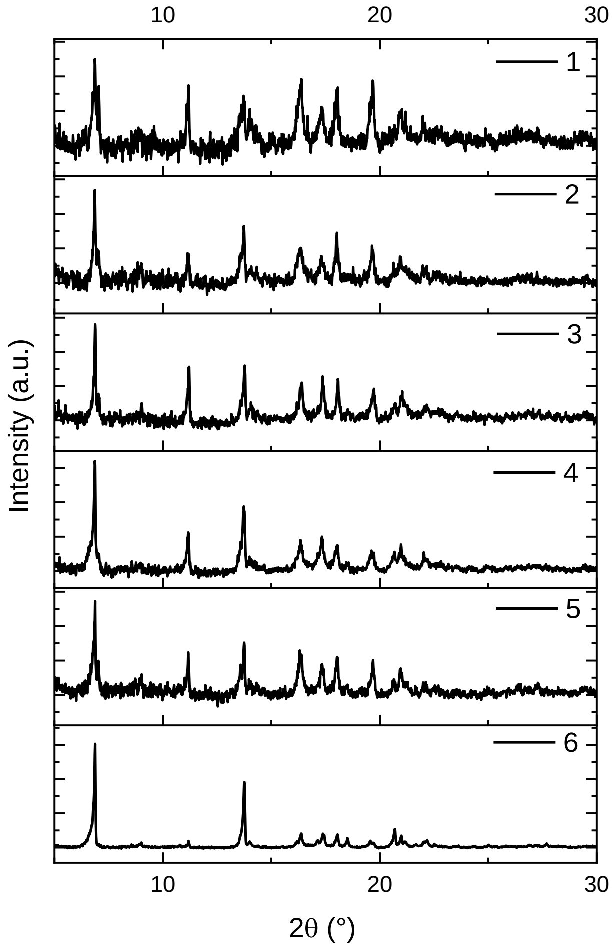

Figure S9. XRD patterns of the crystallized samples at different Reynolds numbers and pH. Samples are numbered according to Table 3.

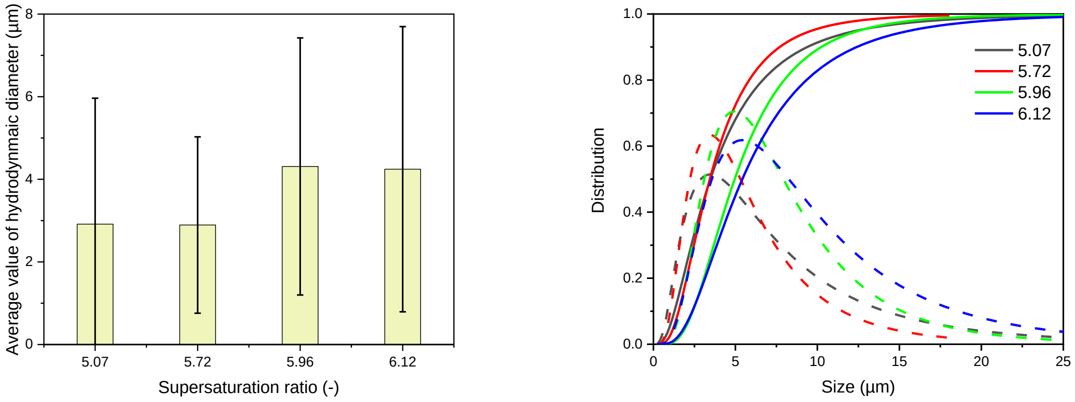

Figure S10. Average values (left), differential and cumulative size distributions (right) of hydrodynamic diameters from LUMiSizer experiments depending on the supersaturation ratio (5.07 – 6.12).

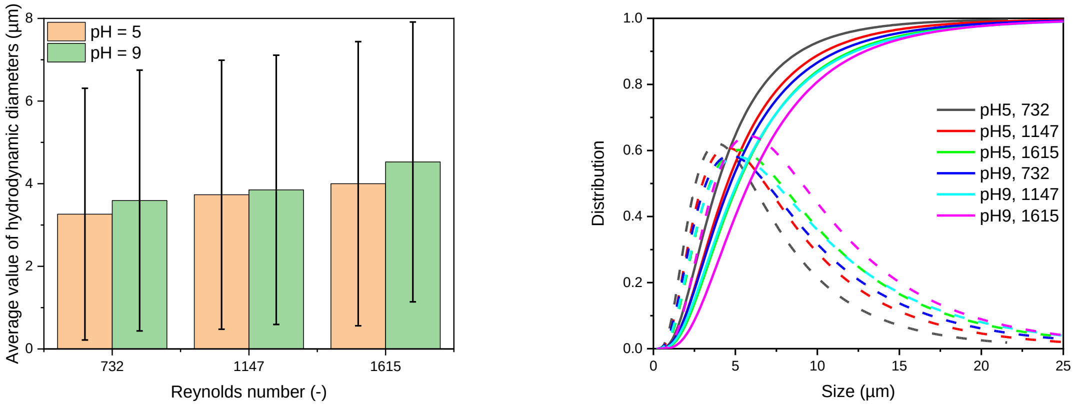

Figure S11. Average values (left), differential and cumulative size distributions (right) of hydrodynamic diameters from LUMiSizer experiments depending on the Reynolds Number (732 – 1615).

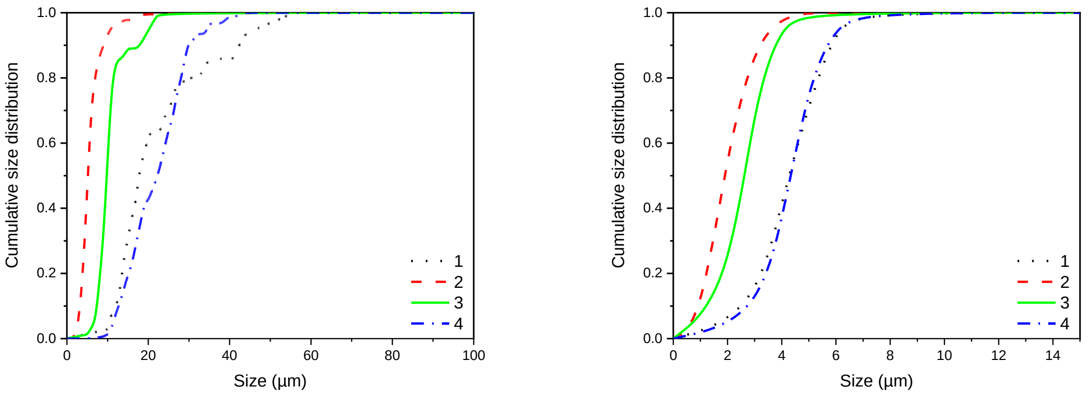

Figure S12. Cumulative size distribution functions based on the analysis of light microscopy images. The particle length (left) and the particle width (right) are illustrated separately. More than 100 particles have been counted for each curve. Crystallized samples at different supersaturation ratios (5.07 – 6.12) are numbered with 1 – 4 from low to high supersaturations.

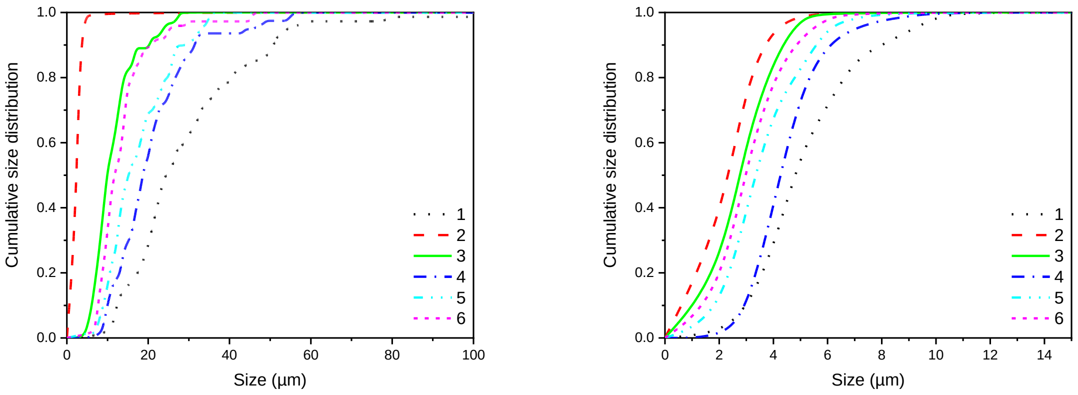

Figure S13. Cumulative size distribution functions based on the analysis of light microscopy images. The particle length (left) and the particle width (right) are illustrated separately. More than 100 particles have been counted for each curve. Crystallized samples at different Reynolds numbers and pH are numbered with 1 - 6. Samples are numbered according to Table 3 (pH 5, 732; pH 5, 1147; pH 5, 1615; pH 9, 732; pH 9, 1147; pH 9, 1615).

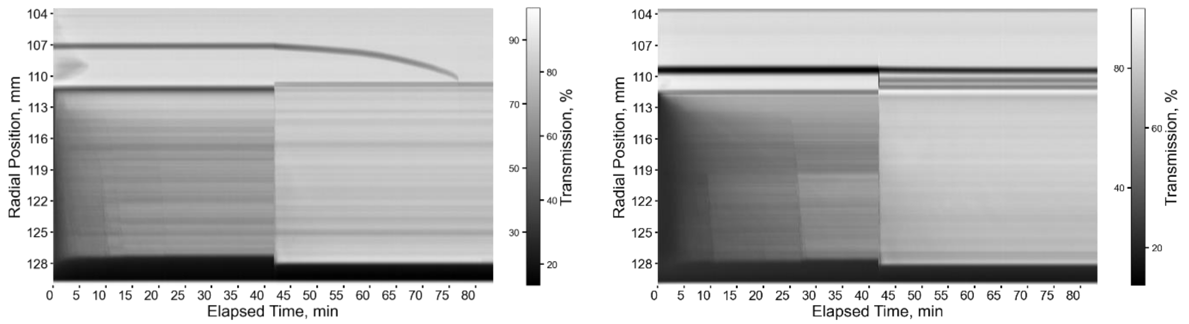

Figure S14. Transmittogram of the sample crystallized at supersaturation ratio 5.07 (left panel) and 5.72 (right panel) respectively. Sample measurement was performed using analytical centrifugation at a constant time interval of 5 seconds, and varying speed from 200 rpm to 500 rpm.

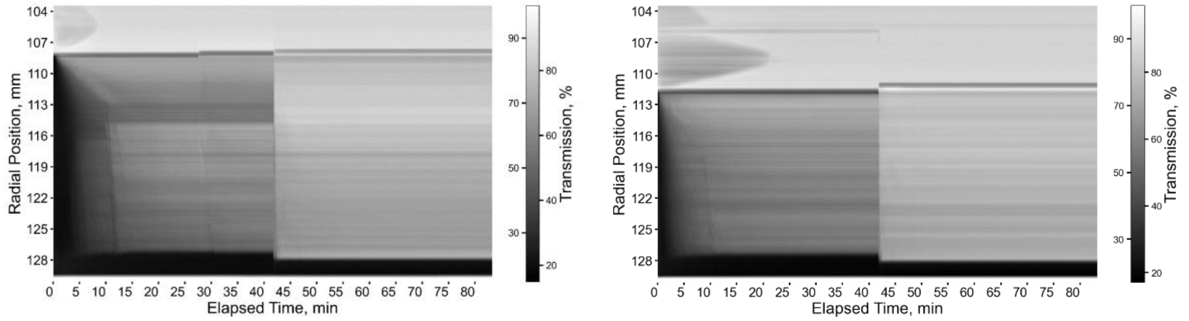

Figure S15. Transmittogram of sample crystallized at supersaturation ratio 5.96 (left panel) and 6.12 (right panel) respectively. Sample measurement was performed using analytical centrifugation at a constant time interval of 5 seconds, and varying speed from 200 rpm to 500 rpm.
